# Vesicoureteral Reflux: Where Have We Been, Where Are We Now, and Where Are We Going?

**DOI:** 10.1155/2008/459630

**Published:** 2008-10-08

**Authors:** Gordon A. McLorie

**Affiliations:** School of Medicine, Wake Forest University Baptist Medical Center, Winston Salem, NC 27157, USA

## Abstract

We present a retrospective review of the scientific and clinical advances, extending over four decades, which have linked vesicoureteral reflux, with renal injury, and urinary tract infection. We have traced the original studies, coupled with advances in technology which led to the awareness, and ability to detect and diagnose the problems early in childhood. These advances progressed through clinical studies which defined the epidemiology of both reflux and urinary tract infection. Along with these diagnostic advances, there were numerous surgical developments, which allowed progressive improvements in the outcomes and effectiveness of a variety of treatment modalities. All of this literature leads us to the current era, when several clinical trials are currently underway in an effort to more fully define the most efficacious and safe methods to treat vesicoureteral reflux and associated urinary tract infection.

Vesicoureteral reflux may have been the major catalyst for the development of the subspecialty of pediatric urology, now approaching a milestone in North America, with the soon-to-be awarding of a certificate of special competence. How did this happen?

In the first textbook of Urology in Childhood, 1974, Dr. Innes Williams included a chapter on reflux, in which his opening sentence states “the problem of reflux has occasioned more controversy than any other topic in pediatric urology” [1]. I submit in writing this article the view that this situation has changed very little to this day, more than 30 years later.

Reflux was recognized very early, as an abnormal function of the ureterovesical junction, but it was Hutch who recognized it in association with neurogenic bladder, in the spinal injured patients, and who linked the reflux to the renal injury in those patients [2]. Reflux was subsequently demonstrated in some pediatric patients with UTI, but there were several studies which showed that reflux was not present in normal infants. These data were brought forward because of the ready availability of voiding cystourethrography—we now assume that these studies are routine and customary—whereas in 1960s and 1970s they were neither available technically, nor did many imagers have any of the facilities or skills that are now standard of care throughout the world.

The next milestone was the recognition that vesicoureteral reflux was associated with urinary tract infections, but also that it occurred as a primary defect in children. Prior principles had shown reflux to be associated with other congenital anomalies or defects such as neurogenic bladder. Hodson and Edwards [3] described a relationship between urinary tract infections and reflux, and further investigators demonstrated this to be present in a significant number of children with recurrent pyelonephritis [4]. These findings led physicians and surgeons to recognize the importance of UTI as a cause of both pyelonephritis and as an extension of this to recognize relationship between chronic scarring and end-stage renal disease, and UTI with reflux. Kunin (1970) published data showing the prevalence of UTI in school-age children. The scene was set for the imposition of two forms of therapy which emerged as the science of the day—antibiotics for gram negative bacterial infections, and surgery for vesicoureteral reflux.

The 1970s witnessed the emergence of antibiotics, including aminioglycosides, chloramphenicol, and cephalosporins, which proved effective in the treatment of sepsis and pyelonephritis caused by gram negative organisms. Although one of these proved myelotoxic and was removed from use, the others continued to be employed more frequently, and further refinements both improved their efficacy and reduced their toxicity. Along with the readily available treatment modalities, the recognition of UTI as an important cause of sepsis in the neonate and young infant became a more common diagnosis. In this era, the differential diagnosis fever in an infant included meningitis which was much more common as a cause of fever and sepsis in infants' than is now the case. Thus, the subsequent investigation of UTI, with personnel and equipment to carry out effective cystograms, led to the diagnosis of vesicoureteral reflux in increasing numbers. Parallel with the growing frequency of the diagnosis of reflux was a growing experience and expertise in the surgery of reflux. Politano and Leadbetter [5] described an effective operative procedure which could achieve successful treatment with relatively minimal morbidity—this became widely utilized in North America, while the Lich Gregoir extravesical techniques [6] were more widely used in Europe. Following upon these successes, Paquin [7], Glenn and Anderson [8], and finally Cohen [9] improvements and modifications of ureteroneocystostomy are resulting in their wide utilization throughout the world in 1980s. The AAP section of urology was started in this period, and the specialty of pediatric urology emerged as a recognized specialty, dedicated to the treatment of children with congenital defects of the genitourinary system.

Dr. John Duckett and a dedicated group of colleagues bridged the gap between pediatric urologists and pediatric nephrologists, in both Europe and North America, to formulate a prospective study to test the hypothesis of the best treatment for vesicoureteral reflux. The international reflux study was born and completed, with publications in 1992, which answered some questions, but left many more unanswered. It was apparent that surgical correction of reflux was feasible, safe, although inconsistent in the complication rates at varying centers. Similarly, it was apparent that reflux would resolve spontaneously. Thus, the most optimal treatment was uncertain. The outcomes measured were primarily renal scarring, but other features of the “disease” became more confusing—was the renal scarring pre-existent, or solely the result of the reflux, or of the UTI? Although dysfunctional voiding was an exclusion factor, the study concluded that 15% of children did have dysfunctional voiding. Was this now to play a part in the treatment of the recurring UTIs? Was the reflux actually a factor in the UTIs, since even after the correction of reflux, persistence of UTIs occurred? Many questions were answered, but many more remained.

In this era of excitement and involvement in the international reflux study, a new player emerged as O’Donnell and Puri [10] published data in 1984, showing that the cystoscopic injection of Teflon paste into the subureteric space could result in the resolution of vesicoureteral reflux. Following the rapid popularization of this technique, mainly in Europe, it was disclosed by researchers in USA [11] that Teflon could potentially be absorbed, and migrate to other areas of the body, including the brain and lymphatics. These data, combined with speculation and fear that leaked Teflon, leaked from prosthetic implants could be a potential cause of autoimmune disease, led the Federal authorities in USA to insure that the subureteric injection of Teflon would not be approved in North America. Nonetheless, a new debate had been born, centered on the child with UTI and vesicoureteral reflux. At meetings, becoming more populated with well trained and proficient pediatric urologists from around the world, debates became heated, stimulating, and amusing. Three of our greatest leaders, each a proponent of either open surgical correction, observational treatment alone or subureteric injection (Duckett, Ransley, O’Donnell), led the assemblies in ever increasing circles of confusion and varied convictions.

Two new pieces of data were added to the continuing puzzle; the emergence of antenatal ultrasound, which showed hydronephrosis in up to 1% of fetuses, and the publication by Noe [12], that vesicoureteral reflux could be shown in up to 25% of siblings who were diagnosed with reflux. The groups of children with reflux diagnosed on the basis of either antenatal hydronephrosis and subsequently diagnosed reflux (20% of those with hydronephrosis), and also those diagnosed on the basis of sibling screening led to an ever increasing population of children with reflux.

Perhaps the latest piece of the technology puzzle, was added by Läckgren et al., who published data on a newer substance, dextranomer/hyaluronic acid copolymer (Dx/HA) [13], which unlike other alternates to Teflon, proved to be durable, effective, and safe. It was approved for use in the USA and Canada and is now widely utilized around the world.

Antibiotic prophylaxis, the nonsurgical treatment modality used throughout all these decades as an alternate to surgical therapy, has now also come into dispute. The emergence of resistant strains of gram negative bacteria is growing, and possibly based on the widespread generic use of many antibiotics, a global increase in methicillin resistant staph aureus (MRSA) is posing serious challenges to treatment of infants with sepsis.

A new multicenter trial is now opened for recruitment in the United States and Canada (RIVUR), funded by the NIDDK, which will randomize children, presenting with UTI, and reflux between treatment with prophylactic antibiotics, and with observation alone [14]. The primary end point is the recurrence of UTI, with secondary end point being the development of renal scar. A similar study is ongoing in France.

We have come full circle, starting with a new diagnosis—reflux, previously unrecognized, which was assumed to be a cause of recurrent uti, and renal scarring, through three decades of evolving developments in technology and science showing a myriad of ways in which we could cure the reflux. Over 25 years ago, Dr. JR Woodard, a world leader of the time, stated “As one looks back over the last 30 years of reflux history, it is ironic that urologists have become so expert at its surgical correction before understanding much about its natural history and true clinical significance” [15]. We now dwell in a world where we STILL question whether the reflux itself is the major problem, or just an easily diagnosed and treated cofactor. Hopefully, the rigors of current science, based on prospective and randomized data, will answer some of these ongoing questions and allow us to treat the children, whom we treat, with the best, safest, most cost-effective, and noninvasive methodologies available to achieve our health-related aims. I believe these aims continue to be the effective treatment and prevention of UTI and the prevention of renal injury.
